# Understanding the impacts of medical tourism on health human resources in Barbados: a prospective, qualitative study of stakeholder perceptions

**DOI:** 10.1186/1475-9276-12-2

**Published:** 2013-01-05

**Authors:** Jeremy Snyder, Valorie A Crooks, Leigh Turner, Rory Johnston

**Affiliations:** 1 Faculty of Health Sciences, Simon Fraser University, 8888 University Dr, Burnaby, BC, V5A 1S6, Canada; 2 Department of Geography, Simon Fraser University, 8888 University Dr, Burnaby, BC, V5A 1S6, Canada; 3 Center for Bioethics and School of Public Health, University of Minnesota, N504 Boynton, 410 Church St. E, Minneapolis, MN, 55455, USA

## Abstract

**Background:**

Medical tourism is a global health practice where patients travel internationally with the intention of receiving medical services. A range of low, middle, and high income countries are encouraging investment in the medical tourism sector, including countries in the Caribbean targeting patients in North America and Europe. While medical tourism has the potential to provide economic and employment opportunities in destination countries, there are concerns that it could encourage the movement of health workers from the public to private health sector.

**Methods:**

We present findings from 19 semi-structured interviews with stakeholders across the public health care, private health care, government, allied business, and civil society sectors. These interviews were conducted in-person in Barbados and via phone. The interview transcripts were coded and a thematic analysis developed.

**Results:**

Three themes were identified: 1) Stakeholder perceptions of the patterns and plans for health human resource usage by current and planned medical tourism facilities in Barbados. We found that while health human resource usage in the medical tourism sector has been limited, it is likely to grow in the future; 2) Anticipated positive impacts of medical tourism on health human resources and access to care in the public system. These benefits included improved quality control, training opportunities, and health worker retention; and 3) Anticipated negative impacts of medical tourism on health human resources and access to care in the public system. These impacts included longer wait times for care and a shift in planning priorities driven by the medical tourism sector.

**Conclusions:**

Stakeholders interviewed who were connected to medical tourism expansion or the tourism sector took a generally positive view of the likely impacts of medical tourism on health human resources in Barbados. However, stakeholders associated with the public health system and health equity expressed concern that medical tourism may spread inequities in this country. The mechanisms by which observed negative health equity impacts in other countries will be avoided in Barbados are unclear. Continued study in Barbados and comparison with the regulatory frameworks in other countries is needed to help enhance positive and mitigate negative impacts of medical tourism on health human resources in Barbados. These findings will likely have import for other Caribbean nations investing in medical tourism and beyond.

## Background

‘Medical tourism’ describes the planned pursuit of privately-purchased medical services by patients outside of their domestic health systems [1-4]. In adopting this definition, we intentionally exclude other forms of international medical travel that are often conflated with medical tourism. As such, cross border care (i.e., arrangements between geographically proximate health systems where formal referral and/or payment networks span international borders), emergency medical care accessed by tourists who become ill or injured while traveling abroad and require medical attention, and routine medical services used by expatriates during their stay outside their home country are not forms of medical tourism [5,6]. The global medical tourism industry has undergone significant growth in the past decade, drawing patients from all over the world to medical facilities located in every global region [2,6,7]. Facilities in these regions are being built, renovated, and staffed with a full spectrum of health human resources in attempts to attract these patients, often competing with one-another for clients [8-10].

The push by national governments to develop local medical tourism industries is driven by numerous, interrelated benefits believed to be associated with this sector. Government officials often state that exporting health services helps to diversify economies and attract foreign investment, enhance the development of technically advanced and specialized medical services, and retain health workers who might otherwise emigrate to other nations for higher wages or more satisfying work [11,12]. Critics of medical tourism counter these claims with assertions that the allocation of resources towards the provision of medical care for foreign patients can increase care costs for local patients due to increased demand, incentivize the development of tertiary health services that might draw public resources away from needed primary health care, and encourage internal migration of health workers from the public to the private sector [9,13,14]. The debates between the merits of these two positions are contested and ongoing as they draw heavily on speculation due to there being little empirical evidence available to support the claims made by either group. While there is some limited evidence concerning the impacts of medical tourism on public health and health human resources, research addressing this topic is limited. Therefore, there is a need to develop an understanding of the positive and negative impacts on health equity of the medical tourism industry on this particular dimension of health systems in destination nations [12,15].

Countries and territories within the Caribbean are becoming increasingly significant participants in the global marketplace for health services, and the medical tourism industry specifically. Cuba, the Dominican Republic, and Costa Rica are already well-known destinations for medical tourists [16-19]. In the Cayman Islands and Jamaica, health care facilities are being built with the intention of providing medical care both to local citizens and medical tourists [18,20-24]. It appears that these countries will soon become meaningful players in the global medical tourism industry. Likewise, Barbados, a small English-speaking country with a long history of attracting leisure travellers from Canada, the United States (US), and elsewhere, is taking steps to transform itself into a destination for medical tourists [25-27]. For example, building upon two existing facilities that already attract medical tourists, the Bajan government solicited bids to redevelop a former hospital site and in 2011 signed a Memorandum of Understanding with American World Clinics (AWC), a US-based company that plans to construct a new 70 bed hospital which will primarily treat medical tourists [28]. The establishment of this agreement suggests that Barbados’ medical tourism industry will undergo significant transformation in the next years.

While the medical tourism industry in Barbados is modest in size and significance relative to those that exist in other Caribbean nations, the industry is poised to undergo significant transformation due to development of the new above-mentioned AWC facility [29]. In 2014, AWC intends to open a multi-specialty hospital primarily serving medical tourists. This hospital will offer a full range of elective surgical and diagnostic procedures [30]. AWC orients its services primarily to medical tourists, with local Bajans only providing a small share of its intended client base. Additionally, AWC is employing a staffing model where international surgeons are being recruited to treat patients on a part-time, membership-style basis [31]. AWC executives anticipate recruiting physicians from the US, Canada, United Kingdom, Europe, and the Middle East [32]. The leasing agreement with the Bajan government commits the hospital to employing Bajan nurses, technicians, and other hospital staff [33]. The purpose of this agreement is to increase local employment, help mitigate the ongoing emigration of health human resources, and ensure that there is training and technology transfer between AWC and the wider community [34]. Given the ambitious size and scope of AWC, in the future this hospital will likely have the greatest impact of all medical tourism providers on the health system, including health workers, in Barbados. In Table 1 we provide a succinct overview of these providers.

**Table 1:** Existing and currently planned medical tourism facilities in Barbados

**Facility Name**	**Date Opened**	**Procedure Focus**	**Features**
Barbados Fertility Clinic	2002	In-vitro fertilization	Only Joint Commission International accredited fertility centre in the Caribbean
Sparman Clinic	2009	Cardiac evaluation	Wellness centre and pharmacy affiliated with clinic
American World Clinics	2014 (expected)	Comprehensive care including surgical, specialty, and dental services	Biotech development and a sports rehabilitation centre are also planned

Barbados has a strong public health care system, comprised of the Queen Elizabeth Hospital, the one major tertiary care centre, and eight primary care clinics distributed across the nation. The public system represents 60% of health care spending in the country, though the proportion of public spending is falling [35]. The private health sector is represented by a small inpatient hospital and a private primary care clinic which, taken together, offer a limited number of surgical specialties and diagnostic services [36]. While Bajans often seek care from both the public and private systems, access to insurance coverage and the private system is more challenging for those with lower incomes [35]. Barbados’ health system faces significant pressures with regard to maintaining an adequate number of health workers in its public sector. In particular, the country has struggled to retain nurses. While the ratio of health providers per 1000 population is not at a critically low level as per World Health Organization standards [37], the ongoing loss of nurses continues to negatively impact the ability of the national health system to deliver effective care [35]. Nurse emigration also represents a significant loss of public investment given the public funding that supports the local training of these health workers [38]. As a response to these shortages, Barbados has increased training opportunities for both physicians and nurses, though shortages of specialists remain [35].

Couched by an awareness that Barbados has ongoing health human resources retention challenges and a new mandate to grow its medical tourism sector, in this article we examine stakeholders’ perceptions of the potential impacts of Barbados’ growing medical tourism sector on the country’s health workers. This article provides a novel contribution to the medical tourism literature in that we offer a forward-looking perspective on these perceived impacts in a country that is on the precipice of substantially growing its medical tourism industry. This analysis thus serves as a baseline for understanding what these impacts are thought to be, wherein this baseline can be revisited in the future as the country’s industry has grown, or continues to grow. We offer some of the first empirical insights about Barbados’ medical tourism sector, drawing on insights shared by 19 interviewees across the public health care, private health care, government, allied business, and civil society sectors. Barbados’ Minister of Health has stated that AWC administrators and Bajan health system officials will coordinate their efforts and policies to mitigate whatever negative effects the hospital might have on domestic public health care capacity and health equity [39]. However, there is little publicly available information concerning exactly how effects upon health human resources will be identified and managed and, therefore, reason to be concerned whether negative impacts on health equity in Barbados will be avoided. We see the current article as serving to stimulate dialogue about this very issue through identifying areas in which impacts are expected and potential mitigation actions.

## Methods

The purpose of this qualitative study was to identify the positive and negative forward-looking (i.e., anticipated or future) health equity impacts of Barbados’ growing, yet nascent, medical tourism sector. Our study was guided by case study methodology, which requires a focus on understanding a phenomenon within the context in which it is happening [40]. Often multiple methods are required in order to fully understand such context [41]. For the current study our methods involved conducting one-on-one semi-structured interviews with stakeholders in Barbados’ medical tourism sector and also conducting observational site visits to public and private health care facilities on the island. We use the term stakeholders to characterize individuals who, in their professional positions, have some degree of involvement in promoting, developing, enhancing, resisting, and/or creating policy regarding Barbados’ medical tourism sector.

### Recruitment

Prior to undertaking recruitment, ethics approval was sought and obtained from the Office of Research Ethics at Simon Fraser University. Following obtaining this approval, medical tourism stakeholders were sought to participate in face-to-face interviews during our team’s site visit to Barbados in April, 2011 using multiple strategies. We aimed to focus on breadth in recruitment, wherein we wanted representation from a diversity of agencies across the target sectors, rather than exhaustively interviewing multiple people from the same agencies.

We extensively reviewed media coverage of the island’s medical tourism, tourism, business development, and health care sectors, as well as online websites and blogs, in order to identify stakeholders across the public health care (e.g., health care administrators and directors, physicians), private health care (e.g., medical tourism facility operators), government (e.g., tourism, development, and health care representatives), allied business (e.g., hotels and tourism operators, health tourism sector), and civil society (e.g., non-for-profit organizations, health system reform advocates, media) sectors. Through this review process a total of 47 potential interviewees were identified. Prior to arriving on-site in Barbados these individuals were contacted directly via email with an invitation to participate in a face-to-face interview while we were on-site in Barbados. We also asked these individuals to suggest other stakeholders whom we should consider interviewing or to pass our invitation to participate in an interview along directly. This strategy resulted in a total of 11 interviews being scheduled prior to our arrival in Barbados. Through conducting these interviews and on-site facility visits while in Barbados additional stakeholders were identified. We conducted 8 interviews with these additional stakeholders, 5 of which occurred by phone from Canada upon return from Barbados (wherein their scheduling could not be accommodated during our time in Barbados or they were not located in Barbados). All participants were guaranteed anonymity.

### Data collection

As noted above, data collection was undertaken in the spring of 2011. Data collection ceased when no new target sectors or agencies for recruitment could be identified. Interviews were conducted in person or over the phone by the first author, allowing for great consistency in the data collection process. Interviews lasted between 30 min and 1.5 hours and were undertaken after a signed consent form was received. At the end of the interviews participants were given a $50 honorarium to acknowledge their important contribution to research.

The interviews were semi-structured, which means that participants were able to share insights that they were not explicitly asked about [42]. The semi-structured guide was created using input from all authors under the leadership of the first author. The guide was organized into four sections: (1) participant background; (2) medical tourism in Barbados; (3) public perceptions of medical tourism in Barbados; and (4) impacts of medical tourism in Barbados. A total of 30 questions were asked. Table 2 contains a selection of the interview questions that were posed. As shown in Table 2, some questions had sub-questions in order to more deeply probe particular issues.

**Table 2:** Selected interview Questions from the semi-structured guide

**Section of interview guide**	**Selected questions**
Background	What is your professional role and how long have you held it?
Medical Tourism in Barbados	What regulations are in place for the medical tourism industry in Barbados?
	Do you know of any plans to add or change these regulations?
Public Perceptions of Medical Tourism	Is there support for expansion of the medical tourism industry in Barbados?
	If so, how is this expressed?
	What forms of expansion is there support for?
	Are there types of expansion that are especially controversial?
Impacts of Medical Tourism in Barbados	Has (or will) medical tourism served as a source of employment for Bajans?
	What sectors have benefitted from this?
	Are you aware of (or do you anticipate) any positive effects of the medical tourism industry on Barbados’ economy?
	If so, what are they?
Are you aware of (or do you anticipate) any negative effects of the medical tourism industry on Barbados’ economy?	If so, what are they?

### Data analysis

All interviews were digitally recorded and then transcribed verbatim. Upon completion of data collection, the transcripts were entered into N7, which is a qualitative data management program. Coding using inductive and deductive strategies then took place following scheme development. Our coding scheme was developed in multiple steps. First, the lead author assigned transcripts to be reviewed in full to each author. Following independent transcript review a teleconference was held to discuss emerging issues and ideas for analytic foci. Second, the lead author compiled a proposed coding scheme that accounted for the issues of interest to the team. The scheme was circulated for feedback. Changes were implemented by the first author following consensus from the team. Third, an external researcher who was not part of the team reviewed the scheme and a selection of the transcripts in order to confirm the parameters of each code and thus the reliability of the scheme. This step added rigour to our process, as did our continual use of investigator triangulation throughout the coding process [43]. Fourth, the external researcher coded the data in close consultation with the first author.

**Figure 1:**
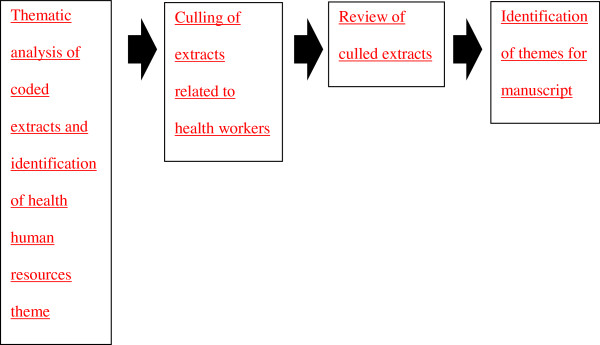
Identification of Themes.

Following the completion of coding, extracts were reviewed in conjunction with the existing literature on the health equity impacts of medical tourism as a way of conducting a thematic analysis [44]. Through this review process an analytic focus on health human resources was developed. Although only one question on the interview guide *directly* probed issues of health human resources, much discussion of this health system resource transpired throughout the interviews. Extracts pertaining to discussion of health workers in the stakeholder interviews were culled from the dataset and saved as a separate document. These extracts were circulated among the authors for review and discussion of emerging themes, particularly in light of discussion of this issue in the existing literature. Thus, the extracts were independently reviewed to identify patterns and outliers in this part of the dataset [44]. Three themes that best characterize participants’ views on the forward-looking impacts of medical tourism development on health workers were identified and confirmed through this review process, which are expanded upon in the following section. Verbatim quotations included in the results section were selected for inclusion from the extraction document by the first author and confirmed for inclusion by the remainder of the team through the processes of review and revision. Figure 1 visualizes this process.

## Results

In total, 19 stakeholders in Barbados’ medical tourism sector were interviewed. 2 of these participants were based in the public health care sector, while 7 were based in private health care, 4 in the government, 3 in the allied business, and 3 in the civil society sectors. The stakeholders with whom we spoke had mixed views of the quality of care offered in the island’s public health care system and the access to this care by Barbados’ residents. While there was a general attitude that health human resources levels and training in the country were improving, there remained a feeling that enhancing levels and training are a crucial factor in improving the care available to the local population and that development in the medical tourism sector could, but need not, threaten this. Not surprisingly, members of the private health sector and allied businesses were generally more optimistic of the potential for positive impacts of medical tourism on health human resources in Barbados than were members of the government and civil society.

In what follows we present the findings of our thematic analysis, focusing on three thematic issues that emerged from the interviews as they relate to health human resources. These issues are: stakeholder perceptions of the patterns and plans for health human resource usage by current and planned medical tourism facilities in Barbados; anticipated positive impacts of medical tourism on health human resources and access to care in the public system; and anticipated negative impacts of medical tourism on health human resources and access to care in the public system.

### Patterns of and plans for health human resource usage by medical tourism facilities

Stakeholders indicated two major motivations for hiring local health workers into the private medical tourism sector. The first motivation, from the perspective of medical tourism companies, is to maximize cost savings for their businesses. When compared to salary expectations for health workers from elsewhere in North America, some local health workers receive significantly lower wages. As one stakeholder from the private healthcare sector explained, foreign investors in the medical tourism sector will find that “*in Barbados I can pay that person [a nurse] more than he or she already makes in the country but I can get that person for less than half the cost [of what the investor would have to pay in his/her home country], so …when you multiple that out across several hundred employees it’s a tremendous costs savings.*” A second motivation for hiring local health workers is to increase employment opportunities in Barbados. For example, according to one government stakeholder, the hiring of health workers is requirement of reaching an agreement with the Bajan government to get approval for a new medical tourism facility. Thus, the government does not see the hiring of local health workers by the medical tourism sector, at least in certain positions, as problematic. Rather, it regards expansion of Barbados’ medical tourism industry as a strategy for developing additional employment opportunities. It was perceived as important that these opportunities would be outside the tourism sector, on which Barbados is highly dependent, offer appealing working conditions, and provide a basis for retaining skilled health workers.

At present, use of existing medical tourism facilities by the local population in Barbados is thought to be quite limited. This is not surprising as the sector is very small, currently focusing on the Barbados Fertility Centre. At this internationally-accredited site, which focuses on fertility treatments for international patients and is highly visible outside Barbados, the hiring of Bajan health workers is proportionally very low. This is due in part to the highly specialized nature of fertility treatment, for which there is limited training offered in Barbados, and the small size of the island’s population is unlikely to ever support training in fertility treatment. As a private health sector stakeholder noted, “*…it's such a niche area that they're not going to come out of the locally trained [health worker labour pool].*” There was little concern among the stakeholders about this practice and no suggestions put forth that the island enhance its specialized medical training in specialties offered in existing medical tourism facilities.

Stakeholders agreed that future hiring practices in the medical tourism sector will have a greater impact on local health worker availability and the public health system than it currently does. This is the case in part because of the large scale of the AWC facility, which will “*need to employ several hundred people*.” It is anticipated that in the future, health workers such as orderlies, nurses, and non-specialized workers will need to be hired locally. As one private health sector stakeholder described it, hiring will start with “*kitchen, restaurant, grounds maintenance, laboratory testing, etc.*” until it is demonstrated that there are not local shortages in other, more specialized areas. There is reported to be potential for local health worker training opportunities to be increased in response to expanding demand from the private sector. A public sector representative reported: “*from what I’m understanding in the US and Canada there’s continued professional development across the board … they [Bajan health workers] have to keep on training and retraining in order to have access to these export markets.*” Opportunities to work in the private sector will be extended to local physicians as well, assuming they are able to meet the training and certification standards for these positions that may be required by international accreditation agencies (e.g., Joint Commission International). This potential expansion of the degree and range of usage of local health workers in the medical tourism sector will mark a significant departure from past and current practices.

### Perceived positive impacts of medical tourism on health human resources and care access

The medical tourism sector in Barbados was perceived as having several forward-looking potential positive impacts on health human resources levels as well as on the quality of care in the public health system. Stakeholders indicated that training limitations had historically presented a barrier to hiring in the private medical tourism sector. Expansion of this sector is thought to present new training opportunities for local health workers and, consequently, increased access to specialty services by the local population. A public sector representative raised the possibility that the international accreditation and training standards required by an expanded private sector would put pressure on local training to improve:

"…it may even help to keep their [health workers in the medical tourism sector] standards up to date because…there’s continued professional development across the board, so if… they have to keep on training and retraining in order to have access to these export markets, that’s also a very positive benefit of medical tourism."

It was also thought that a return to the public sector following employment in the medical tourism sector could have system benefits: “*It [working in the medical tourism sector] has also allowed for experience and practice in medicine, doing different things…persons go out and get specialized and come back and they’re more capable.*” Linked to this and the increased training opportunities presented by the private medical tourism sector, it was thought that the overall quality of medical care in Barbados could improve in response to expansion of the medical tourism sector.

Historically, attaining sufficient numbers of health workers in Barbados’ public sector was challenged by out-migration. Expansion of the medical tourism sector is perceived by some stakeholders as potentially helping with retention of health workers by offering higher paying jobs at local, private health care facilities. Medical tourism presents a new form of migration: that of the move from public to private practice among local health human resources. This internal migration of health workers impacts not only the quality of care offered in the health system but was felt by a public sector representative to create a financial burden due to the ongoing need to invest in training of health workers, and nurses in particular: “*we just are challenged cause we don’t have enough nurses here, we are importing our nurses ourselves and they are leaving and it costs a lot of money to train a nurse. And they can never repatriate what we’ve spent and invested on their education.”* Moreover, the medical tourism sector might create more appealing working conditions, thereby further encouraging health workers to practice locally: “*I think that would keep this sector vibrant and also diversifying…I don’t think we’ve embraced a lot of health services, we’re pretty traditional in what we do.*” A stakeholder noted that Barbados is responding to its own nurse retention problems by recruiting nurses regionally from elsewhere in the Caribbean. This was also reported in facility tours we undertook while on-site in Barbados. Thus, should medical tourism ultimately result in retaining health workers who would otherwise out migrate, this would have a positive regional impact by reducing the need to recruit nurses from other Caribbean nations.

### Perceived negative impacts of medical tourism on health human resources and care access

While many stakeholders noted the potential of an expanded medical tourism sector to enhance training opportunities and help retain health workers within the country, several concerns about negative impacts on health human resources and access to health services by the local population were noted. A common concern was that development of the private medical tourism sector, particularly if it draws upon resources from the public sector, will inevitably reduce access by the local population by increasing wait times for care due to many factors including health worker shortages. As one public health sector stakeholder expressed: “*we don’t have spare capacity right now. Because across all surgical areas there are waiting lists, which even if we compare with international standards are not acceptable.*” Orthopaedic surgery wait times was identified as a particular area of concern: “*our average waiting time for orthopaedics for …hip and knees run between twelve to fifteen, sixteen months.*” Thus wait times and their associated challenges will become amplified if system resources become taxed by an expanded medical tourism sector. A related concern is that much current demand for medical care in Barbados is related to trauma. It was thought that trauma patients may be forced to wait longer for care as the business model of medical tourism dictates against asking foreign patients to wait for care: “*you imagine a medical tourist being here, scheduled for surgery today and we have to say sorry, we can’t do it, we have trauma.*”

A concern with expansion of medical tourism in Barbados relates to a perceived shift in planning priorities. In particular, it was suggested that any expansion of the medical tourism sector would likely be driven by the Ministry of Tourism rather than the Ministry of Health, and therefore represent priorities and an ideology inattentive to the health needs of the local population or health human resources demands. This is problematic, according to a public health sector representative, as “*they see it more as a subset of the tourism product without really understanding, is this something we [public health care sector stakeholders] really could, could get in to.*” An ideological focus on raising revenue from medical tourists could undermine equitable access to medical care by the local population:

"There has been discussion openly about medical tourism and the ability to raise revenue… [Y]ou need to look at ethical issues …surrounding, you know, medical tourism, and the key here is access. You know you have to be satisfied that you could provide access to resident population before deciding to treat others."

Thus, the priorities of stakeholders advocating for medical tourism development may differ from and conflict with goals and values of health workers and administrators in the public medical sector.

## Discussion

The stakeholders we spoke with articulated two distinct and competing views regarding the likely impacts of an expanded medical tourism sector in Barbados. Stakeholders in the medical tourism industry and the tourism and economic development sectors within the Barbados government tended to take a positive view of development in this sector, including its likely impact on access to health care within Barbados’ public system. For these stakeholders, medical tourism expansion is an opportunity to create new and better paying jobs for Bajan health workers and stem the out migration of these workers, create new opportunities for training and quality improvement for the Bajan health workforce, and enhance the range of medical offerings on the island, all of which could be accessed by the local population, albeit in the private system; thus, health equity could be left unaffected or even improved by medical tourism. In contrast, stakeholders in Barbados’ public health and civil society sectors commonly raised concerns about the effects of medical tourism expansion on the distribution of trained health workers, as these workers might be enticed to move from the public to private system thereby creating or exacerbating health worker shortages in the public system and harming health equity.

The dominant concerns of the public system and civil society stakeholders we spoke with were focused on health equity, where expansion of the medical tourism sector might promote a private medical sector that draws resources from the public system, including health human resources. This concern mirrors that often voiced by academic critics of the medical tourism sector [9,12]. If this private system is not accessible to all Bajans then the potential forward-looking benefits of medical tourism sector expansion raised by participants from all sectors would likely only be enjoyed by those sufficiently wealthy to access the private system or those health workers who secure employment in that system. Industry and government stakeholders tended to stress aggregate gains in health worker numbers, economic benefits, medical procedures offered, and training levels. The concern voiced by this other group, however, is not necessarily that Barbados will fail to see these gains on aggregate as the medical tourism sector expands. Rather, public sector stakeholders expressed concern that the distribution of these benefits will heighten health inequities within the local population and between locals and international patients.

There is reason for concern, as some stakeholders stressed, that medical tourism expansion in Barbados could have future negative impacts on health equity and even absolute levels of access to care and health worker levels in the public system. This concern is illustrated by a tension found both in the sentiments of the private sector and government stakeholders interviewed and in media coverage of medical tourism in the country. A significant selling point of the AWC hospital is the anticipated expansion of employment opportunities outside the established tourism sector for the local population [33]. However, the stakeholders we spoke with stressed that local hiring will likely be focused on less-skilled positions such as orderlies, janitorial and kitchen services, and laboratory testing. If so, the medical tourism sector has the potential to expand employment opportunities on the island without drawing from a limited supply of highly trained health workers or creating shortages in the public system. At the same time, the stakeholders indicated that the AWC hospital will create opportunities for local physicians to work within the private, medical tourism sector. While these opportunities might not result in physician shortages in Barbados’ public system, such statements reveal that employment of Bajans in the medical sector will indeed extend to skilled health care professionals. Similarly, it was noted by several stakeholders that theatre nurses will not initially be hired from the local population by AWC. However, nurses in other specialties will be hired, raising concern about long-term skilled health worker levels in the public system. Thus, the public to private migration of health workers seen in other medical tourism destinations such as Thailand [45,46], Malaysia [8,47] and India [48,49] could potentially be created in Barbados as well. These comments serve to highlight the extremely complex, multi-dimensional nature of the relationship between health human resources, public health care, and private health care in a country looking to grow its medical tourism sector.

Accompanying the tensions in proposed hiring practices within the medical tourism sector reported above, numerous stakeholders indicated awareness of the potential for an expanded medical tourism sector to negatively impact health human resource levels in the public system and for the need for steps to protect against these impacts. Several stakeholders stated that plans are in place to prevent large-scale hiring of health workers into private medical tourism facilities that are in short supply in the public system. The exact nature of these plans is not yet publicly known. Barbados’ Minister of Health has stated publicly that coordination between the government and medical tourism sector will help ensure that hiring practices do not negatively impact the public sector and that these requirements were included in the Memorandum of Understanding between the government and AWC [39]. What is not clear from this statement is how this will be done, the types of mitigation strategies that will be put in place, how regulation will occur, and if any legislative actions may be forthcoming to regulate hiring of health workers in the private system. This limited transparency and reported non-inclusion of public system voices in the early stages of the AWC development does not mean that the planned steps to protect against public to private health worker migration will be insufficient. Skepticism about these plans is warranted, however, and greater transparency and stakeholder inclusion is needed to ensure that crucial voices in long-term health human resources planning in Barbados’ medical tourism sector are heard.

### Future research directions

Acknowledging that Barbados’ medical tourism industry seems poised for growth, it is important that data be gathered to better understand the impact of this sector on health human resources, as well as other health equity dimensions. Careful study of the development of medical tourism in Barbados can provide insight into the realized effects on health workers in that country and contribute to broader comparative, cross-national analysis of medical tourism. Such research will provide a valuable resource for better managing medical tourism, including mitigating negative impacts and strengthening positive ones. This information, in addition to being of use to stakeholders in Barbados, should have value for similarly-situated Caribbean nations that are exploring the possibility of expanding the medical tourism industry or are in the process of building new facilities such as the Cayman Islands and Jamaica [18,20-22].

In other countries with growing medical tourism sectors, the reported impacts of medical tourism on health human resources have differed depending upon a variety of factors. These documented impacts range from greatly negative to little identifiable impact [8,45,46]. While these early studies of the effects of medical tourism on health workers need to be supplemented with additional research, it is clear from the current study in Barbados that regulatory frameworks, as well as other contextual factors, have a significant influence on what stakeholders perceive are the potential impacts of medical tourism. An important future research direction emerging from this study is to identify the full range of regulatory frameworks being developed in countries looking to establish a medical tourism sector. Identifying common and distinctive elements among these frameworks will help to provide an important knowledge base as countries look to identify legislative strategies for managing the impacts of the medical tourism industry, whether positive or negative.

Gathering the on-the-ground insights of medical tourism stakeholders is crucial to better understanding local interpretations of medical tourism as well as its current and anticipated, or forward-looking, impacts. Combined with careful analysis of policy and legislation, acquisition of quantitative data on the effects of medical tourism on health human resources, continued stakeholder consultation within and beyond Barbados will provide valuable insight into the significance of medical tourism for health systems, which is something that is lacking in empirical insights [7]. Development of a richer, more rigorous evidence base will assist health policy makers and other stakeholders as they weigh advantages and disadvantages of competing for medical tourists in the global health services marketplace.

Based on the findings of this prospective study, we are taking several steps to continue this research. First, we are partnering with local investigators in Barbados with the aim of disseminating our research findings to local stakeholders and better understanding local health human resource needs. Second, we are actively seeking funding to support research aimed at better understanding the impact of medical tourism on health human resources in Barbados. Finally, in our continued research we are assessing the viability of policy responses intended to mitigate any negative and enhance any positive impacts of medical tourism on health human resources in Barbados and other sites. The prospective results contained in this manuscript will thus serve as a basis for continued research into the impacts of and responses to medical tourism expansion in Barbados and elsewhere.

### Wider relevance

This article adds to a growing body of knowledge on the policy development surrounding and impacts of medical tourism in destination countries, especially with regard to health human resources. Consistent with Ormond’s [50] findings in the Malaysian context, policy makers promoting the development of medical tourism in nations with strong public health care systems such as Barbados may have an unsupported belief in the separation of resources between public and private health systems, especially as this relates to growing a medical tourism sector. In light of the findings of the current article and those such as Wilburprastert’s [51], which suggest that medical tourism has a significant impact on Thailand’s health workers for local patients, it is important that health system planners in destination nations maintain a critical approach to creating policies promoting the development of medical tourism. This is particularly true for countries that are in the prospective planning stages, such as Barbados, where policy can be proactive rather than reactive.

The key role of Barbados’ Ministry of Tourism in spearheading the development of medical tourism in the country suggests that our findings may have particular relevance to other tourism-dependent nations looking to grow their medical tourism sectors. As medical tourism development has recently become a popular initiative by tourist destinations, especially amongst countries in the Caribbean, the Bajan example of the Ministry of Tourism planning for medical tourism in relative isolation from the Ministry of Health offers insight into what may be a common approach to sector development. Better policy coordination between Ministries in Barbados and other up-and-coming tourism-dependent destination countries may allow for the development and implementation of medical tourism policies that are better suited to protecting the existing health system from the outset. Once again, such coordination may result in the development of proactive, rather than reactive, policy.

### Limitations

Two main limitations undergird this analysis. First, our use of phone interviews with 5 participants meant that we were unable to observe body language and emotional displays during the course of these interviews. While this is a limitation that has been noted of phone interviewing [52], the cost effectiveness of this data collection method overrode such concerns by permitting increased participation in the study. Second, while a wide range of perspectives was sought in the recruitment process, we ultimately spoke with few stakeholders from the civil society sector as well as few currently practicing health workers (versus health system administrators or policy-makers). As such, the views held by such groups are likely underrepresented in this analysis.

## Conclusions

Given the upcoming expansion of Barbados’ medical tourism industry, the Bajan public health care system is nearing an important crossroad. At present, the medical tourism sector in Barbados is small, involves few physicians and other health care providers, and has had little noticeable impact on health human resources distribution and training. Assuming that completion of the AWC hospital will lead to greater employment of health workers in the private sector, the impact of medical tourism on the Bajan health system will increase. It is possible that this impact will be positive, with new health workers being trained, more physicians and nurses choosing to pursue careers in Barbados, and better employment opportunities created throughout the health system. However, it is possible to envision a much different and far more problematic outcome. If inadequately managed and regulated, benefits of medical tourism might be restricted to the private health system, with new health care resources not being shared with the public sector. An even more worrisome scenario would involve public resources flowing without compensation to the private system. Continued research into and monitoring of the health human resource impacts of medical tourism sector development in Barbados is necessary in order to understand which of these forward-looking scenarios will ultimately take shape in the country and the realized impacts it will have.

## Abbreviations

AWC: American World Clinics; US: The United States of America.

## Competing interests

The author(s) declare that they have no competing interests.

## Authors’ contributions

All authors were in Barbados during the on-site data collection period and participated in daily team debriefings about the interviews, site visits to public and private health care facilities, and emerging analytic and research ideas. JS designed the interview guide, conducted the interviews, led this analysis, and led writing this article. VAC contributed to study design, the process of confirming the interpretation of findings, writing sections of this article, and editing this article. LT provided input into the interview guide, writing sections of this article, and editing this article. RJ provided input into the interview guide, wrote sub-sections of this article, and also edited the article. All authors read and approved the final manuscript.
